# Multimorbidity and statin prescription for primary prevention of cardiovascular diseases: A cross-sectional study in general practice in France

**DOI:** 10.3389/fmed.2022.1089050

**Published:** 2023-01-09

**Authors:** Racha Onaisi, Roxane Dumont, Jennifer Hasselgard-Rowe, David Safar, Dagmar M. Haller, Hubert Maisonneuve

**Affiliations:** ^1^Department of General Practice, University of Bordeaux, Bordeaux, France; ^2^Unit of Population Epidemiology, Division of Primary Care Medicine, Geneva University Hospitals, Geneva, Switzerland; ^3^Faculty of Medicine, Institute of Global Health, University of Geneva, Geneva, Switzerland; ^4^University College of General Medicine, University Claude Bernard Lyon 1, Lyon, France; ^5^Faculty of Medicine, University Institute for Primary Care, University of Geneva, Geneva, Switzerland

**Keywords:** multimorbidity, cardiovascular diseases, primary prevention, statin, general practice

## Abstract

**Background:**

Statins are a first line, evidence-based yet underprescribed treatment for cardiovascular primary prevention. In primary care settings, multimorbidity is a complex situation which makes it difficult to apply prevention guidelines.

**Aim:**

To assess the associations between multimorbidity and prescription of statins in accordance with the 2016 ESC recommendations (“appropriate prescription”), and to identify the factors and conditions associated with these prescriptions.

**Design and setting:**

Cross-sectional prospective study in the French region of Rhône-Alpes among 40 general practitioners and their patients.

**Methods:**

We examined the association between appropriate statin prescription and several patient characteristics, including multimorbidity, using multivariate logistic regression models.

**Results:**

Between August 2017 and February 2019, 327 patients were included in the study. Seventy-four (22.6%) were on statin medication and 199 (60.9%) exhibited multimorbidity, defined as ≥2 diseases. Only 22.5% of eligible patients were prescribed statins for primary prevention. Diabetes was most strongly associated with appropriate statin prescription (aOR 8.10, CI 95: 3.81–17.80). Multimorbidity was not associated with appropriate statin prescription (aOR 1.31, CI 95: 0.54–3.26), except in the presence of diabetes which defined diabetic multimorbidity (aOR 10.46, CI 95: 4.87–23.35). Conversely, non-diabetic multimorbidity was associated with lower odds of being appropriately prescribed a statin (aOR 0.26, CI 95: 0.12–0.56).

**Conclusion:**

Multimorbidity, in itself, does not seem to be a determinant factor for appropriate statin prescription. The latter appears to be determined by a patient’s type of multimorbidity, especially the presence or not of diabetes. Differentiating between diabetic and non-diabetic multimorbidity may be a pragmatic way for GPs to improve primary prevention in a patient-centered and shared decision-making approach.

## 1. Introduction

Statin prescription is a cornerstone of primary cardiovascular prevention. In the primary care setting, multimorbidity is a complex situation which makes it difficult to apply prevention guidelines in daily practice (1–3). This study set out to understand the association between multimorbidity and the prescription of statins as primary prevention in primary care.

The World Health Organization defines multimorbidity as an association of two or more diseases that can impact a person’s health status. Since many people consider this definition insufficient to summarize multimorbidity, numerous definitions can be found in the literature (4–6). Cardiovascular diseases and metabolic diseases, such as diabetes mellitus or obesity, are among the most represented health issues in patients with multimorbidity (7, 8). They are also the most common cause of death in Europe (9). Statin therapy for primary prevention in patients with high cardiovascular risk has been continuously recommended as a first line treatment (10, 11). Yet, despite evidence supporting use for primary prevention in high-risk patients, recent studies conducted in the United States and in Europe have found that statin therapy was prescribed in only 20–60% of eligible patients (12–14).

In primary care, a number of factors come into play when deciding whether or not to treat a multimorbid patient with statins: (i) the underuse of risk assessment scores (15), (ii) the poor applicability of single-diseased based guidelines (2), or (iii) the complex prioritization of health interventions (16–18).

To our knowledge, there is not much data about the associations between multimorbidity and statins under-prescription, and published data is sometimes contradictory (12, 19). For instance, in a recent study performed within the French-speaking population in Canada, multimorbidity was strongly associated with an increased likelihood of using statins for primary prevention (adjusted OR 3.76) (19). Yet, in that study, multimorbidity was assessed by using the number of chronic conditions declared by patients and was not further described. As far as cardiovascular prevention is concerned, the question remains: should asthma or osteoporosis influence statin prescription as much as diabetes and hypertension? We felt that these results needed to be explored further, so as to clarify the conditions that may constitute multimorbidity.

In this view, the aim of this study was therefore (i) to assess the associations between multimorbidity and the expected under-prescription of statins in accordance with guidelines for primary prevention, and (ii) to identify the factors and conditions associated with these prescriptions, in a primary care population.

To reach this aim we conducted a cross-sectional prospective study measuring the statin prescription rate in accordance with the 2016 European Society of Cardiology (ESC) guidelines on cardiovascular disease primary prevention (20), referred as “appropriate prescription” and the factors associated with the appropriateness of the prescription.

## 2. Materials and methods

### 2.1. Study population

We recruited general practitioners (GPs) in the French region of Rhône-Alpes using the snowball method, starting from a convenience sample of eight GPs affiliated within the primary care unit of Lyon University (France). We looked for maximum variation according to the following criteria: age, sex, duration of clinical practice, continuing medical education, type of practice (alone, group, pluri-professional structure), and participation in resident clerkships.

When a GP agreed to participate, a recruitment date was booked with a research assistant. On the day scheduled for the study, the research assistant was physically present in the GP practice. Patients were assessed for eligibility during their appointments with their GP, without modifying their scheduled consultations. Patients who agreed to participate were progressively included in the study until a maximum number of eight patients per GP was reached.

Patients were eligible if they had had their cholesterol levels measured during the preceding year, or had a statin prescription for primary prevention purposes, or presented at least one of the following cardiovascular risks: diabetes mellitus, family history of cardiovascular disease, active smoking, or high blood pressure.

Patients were excluded if they had a personal history of previous or current cardiovascular diseases (myocardial infarction, stroke, transient ischemic stroke, or peripheric arterial disease). They were also excluded if their cholesterol level had not been assessed in the previous 5 years.

### 2.2. Data collection

Data collection took place between August 2017 and February 2019. When eligible patients agreed to participate, a research assistant, or the GPs themselves, completed an anonymous questionnaire collecting sociodemographic data (age, sex, profession, and ethnicity), clinical data (weight, height, systolic blood pressure, cardiovascular risk factors, or diseases associated with cardiovascular risk), patients’ self-perceived state of health, biological data (cholesterol levels, presence of microalbuminuria, and renal function), and current treatments (with a specific item focused on statin intake). The presence of multimorbidity was assessed using a list of 75 chronic conditions (21) which had been created to explore the prevalence of multimorbidity in a primary care population in the Swiss national study on multimorbidity and patterns of chronic conditions (22, 23). We considered multimorbidity to be present when the patients were suffering from two or more chronic conditions on the list.

To assess statin underprescription, we chose to calculate the rate of statins prescribed in accordance with the 2016 ESC guidelines on cardiovascular disease primary prevention, in our work referred to as “appropriate statin prescription.” Appropriate statin prescription was calculated as the rate of patients prescribed statins among the total population of patients eligible for primary prevention with statin therapy. To ensure that the chosen guideline did not induce bias, we first compared the rates of statin therapy prescribed in accordance with ESC 2016 with the rates prescribed in accordance with other guidelines [French HAS 2017 (24), ESC 2019 (11), NICE (25), and AHA 2018/2019 (10)]. These guidelines all define at-risk patients, mainly relying on risk assessment scores, to determine which patients could benefit from statin therapy.

### 2.3. Sample size estimation

We calculated the sample size required for our study using the formula for proportions estimated with a given precision. Based on previous studies (12), we estimated that the maximum appropriate statin prescription rate was 62%. We wanted to be able to provide a 95% confidence interval width of 0.05 for the estimate. The minimum required sample was estimated at 377 patients. Statistical significance was set at a two-tailed *p*-value ≤ 0.05.

### 2.4. Statistical analysis

We compared sociodemographic and medical characteristics of participants with and without statin medication, using Fisher’s exact test, Chi-squared and Student’s *T*-test, as appropriate. Continuous quantitative variables were described by mean and standard deviation (SD) values. Discrete variables and categorical variables were described by frequency and proportion values.

To assess factors associated with appropriate statin prescription, we examined the associations between baseline clinical characteristics and compliance with the ESC 2016, using multivariate logistic regression models, and reported results as odds ratios (OR) and 95% confidence intervals (CI). Patients with statins prescribed according to the guidelines were compared to those not receiving any statin therapy. Several logistic regression models were tested, using two or more chronic conditions and three or more chronic conditions to define multimorbidity, including diabetes or not. Participants with missing data for at least one of the covariates were excluded from the models. All analyses were performed with R (version 4.0.3).

### 2.5. Ethical approval

This is not an interventional study. In line with the applicable French law at the time of the study design and data collection, ethical approval was not required.

## 3. Results

### 3.1. Characteristics of the study population

**FIGURE 1:**
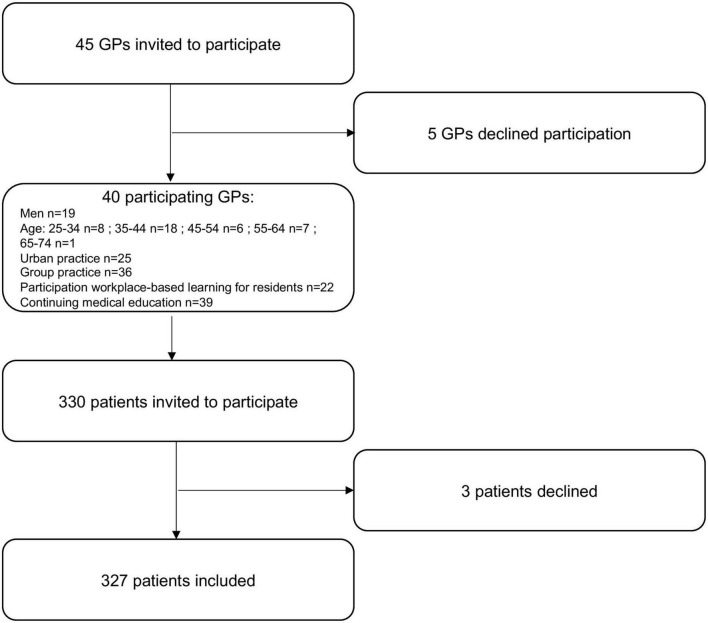
Flow-chart of general practitioners (GPs) and patients inclusions.

Five GPs declined participation (11%). The participating GPs invited a total of 330 patients to participate, three of them declined (0.9%) (Figure 1).

Among the 327 included patients who did not present any previous history of cardiovascular disease, 74 were on statin medication (22.6% of total study population). About half were more than 65 years old [*n* = 99 (30.4%) between 65 and 74 years old and *n* = 67 (20.6%) above 75], and 40.5% were male. More than half of the patients were considered multimorbid (*n* = 199, 60.9%). Table 1 reports the characteristics of the total study population, stratified by statin use.

**TABLE 1:** Characteristics of study population, stratified by statin use (*n* = 327).

	Without statin prescription *N* = 253	With statin prescription *N* = 74	Total *N* = 327	*p*-value^a^
	*n* (%)	*n* (%)	*n* (%)	
**Sociodemographic characteristics**				
Age				0.004
<65	136 (54.0)	24 (32.4)	160 (49.1)	
65–74	67 (26.6)	32 (43.2)	99 (30.4)	
≥75	49 (19.4)	18 (24.3)	67 (20.6)	
Sex				0.010
Male	93 (36.5)	40 (54.1)	133 (40.5)	
Female	160 (63.5)	34 (45.9)	194 (59.5)	
Ethnicity				0.861
White	231 (91.3)	66 (90.4)	297 (91.1)	
Black or African-American	17 (6.7)	6 (8.2)	23 (7.1)	
Other	5 (2.0)	1 (1.4)	6 (1.8)	
**Health status**				
Multimorbidity				
≥2 diseases	137 (54.2)	62 (83.8)	199 (60.9)	<0.001
≥3 diseases	78 (30.8)	42 (56.8)	120 (36.7)	<0.001
Self-perceived health status				0.054
Poor or mediocre	10 (4.3)	0 (0.0)	10 (3.2)	
Good	50 (21.3)	23 (31.5)	73 (23.7)	
Very good or excellent	175 (74.5)	50 (68.5)	225 (73.1)	
**Risk factors for cardiovascular diseases**				
Cholesterol levels				
Total [Mean (SD)]	217 (41)	186 (44)	210 (44)	<0.001
LDLc levels				<0.001
40–70 mg/dL	4 (1.6)	13 (17.6)		
70–99 mg/dL	29 (11.5)	26 (35.1)		
100–114 mg/dL	40 (15.8)	11 (14.9)		
>115 mg/dL	180 (71.1)	24 (32.4)		
HDLc [Mean (SD)]	0.60 (0.17)	0.53 (0.17)	0.58 (0.17)	0.006
≥40 mg/dL	227 (89.7)	57 (77)		0.008
Hypertension	*n* (%)	*n* (%)	*n* (%)	
Receiving a treatment for hypertension	105 (41.5)	49 (66.2)	154 (47.1)	<0.001
Systolic blood pressure level [Mean (SD)]	131.79 (13.77)	131.67 (12.61)	131.77 (13.49)	0.946
Diabetes	*n* (%)	*n* (%)	*n* (%)	
Type 2	27 (10.7)	28 (37.8)	55 (16.8)	<0.001
Type 1	5 (2.0)	6 (8.1)	11 (3.4)	0.027
Retinopathy	1 (0.4)	4 (5.4)	5 (1.5)	0.011
Neuropathy	33 (13.0)	8 (10.8)	41 (12.5)	0.756
Current smoker or former smoker for less than 3 years	51 (20.2)	14 (18.9)	65 (19.9)	0.945
Family history of cardiovascular disease	43 (17.0)	18 (24.3)	61 (18.7)	0.210
Renal function impairment with glomerual filtration rate (GFR) < 30 mL/mn	57 (23.0)	12 (16.2)	69 (21.4)	0.278
Body mass index (BMI)				0.086
<25	110 (45.1)	21 (31.8)	131 (42.3)	
25–29.9	81 (33.2)	31 (47.0)	112 (36.1)	
≥30	53 (21.7)	14 (21.2)	67 (21.6)	
Atrial fibrillation	11 (4.3)	6 (8.1)	17 (5.2)	0.325
HIV infection	6 (2.4)	1 (1.4)	7 (2.1)	0.939

^*a*^Fisher’s exact test, Pearson’s Chi-squared test or Student T, when appropriate.

Patients on statin medication were more likely to be male (54.1 vs. 36.5%, *p* = 0.01), aged above 65 years old (67.5 vs. 46%, *p* = 0.004), and multimorbid (83.8 vs. 54.2%, *p* < 0.001). Among known cardiovascular risk factors, patients on statin medication were more likely to have diabetes mellitus (37.8 vs. 10.7%, *p* < 0.001) or treated hypertension (66.2 vs. 41.5%, *p* < 0.001). There were no significant differences between groups regarding family history of cardiovascular disease or active smoking.

### 3.2. Statins prescription in accordance with guidelines

The number of patients eligible for statin therapy varied between 157 and 281 depending on guidelines. Among the 262 patients for whom an indication for primary prevention statin therapy was found in accordance with the ESC 2016 recommendation, 59 were on medication, representing an estimated appropriate statin prescription rate of 22.5%.

No matter what guideline was taken into consideration, statin therapy for primary prevention was under-prescribed. The highest rate of appropriate statin prescription in our study population was estimated at 32.3% (AHA 2018/2019), with an even lower prescription rate estimated at 19.5% (HAS 2017) (Table 2).

**TABLE 2:** Rates of statin therapy prescribed in accordance with different guidelines.

	ESC 2016 (SCORE)	HAS 2017 (SCORE)	ESC 2019 (SCORE)	NICE (QRISK-2)		AHA 2018/2019 (ASCVD)	
				QRISK > 5%	QRISK > 10%	ASCVD > 5%	ASCVD > 10%
Number of patients eligible for statin therapy (Ne)	262	185	281	264	202	190	127
Number of patients treated as recommended (Nt)	59	36	60	71	63	51	41
Appropriate statin prescription rate according to recommendation (Ne/Nt)	22.5%	19.5%	21.4%	26.9.0%	31.1%	26.8%	32.3%

### 3.3. Association between multimorbidity and appropriate statin prescription

In the univariate analysis, female patients were less likely to be treated in accordance with the guideline than male patients (OR = 0.54 [0.30–0.96]). Being between 65 and 74 years old (OR = 2.74 [1.43–5.34]), being treated for hypertension (OR = 2.93 [1.61–5.50]), exhibiting multimorbidity (OR = 3.19 [1.64–6.74]), were associated with an increased appropriate statin prescription.

Diabetes appeared to be the factor most strongly associated with appropriate statin prescription (OR = 8.00 [4.29–15.19]; *a*OR = 8.10 [3.81–17.80]), including in multimorbid patients. Indeed, in our multivariate models, diabetic multimorbidity was strongly associated with appropriate statin prescription (*a*OR = 10.46 [4.87–23.35]), while non-diabetic multimorbidity was associated with lower statin prescription for primary prevention, despite an indication for the treatment (*a*OR = 0.26 [0.12–0.56]) (Table 3).

**TABLE 3:** Univariate and multivariate analysis of factors associated with appropriate statin prescription according to the 2016 European Society of Cardiology (ESC) guidelines.

	No statin therapy *N* = 261^†^	Appropriate statin therapy *N* = 57^‡^	Crude OR [95 CI]	Adjusted OR [95 CI]
	*n* (%)	*n* (%)		
Female	163 (62.5)	27 (47.4)	0.54 [0.30–0.96]*	0.58 [0.29–1.17]^a^
Age				
<65	135 (52%)	19 (33.3)		
65–74	70 (27)	27 (47.4)	2.74 [1.43–5.34]*	2.45 [1.09–5.64]*^a^
≥75	55 (21)	11 (19.3)	1.42 [0.62–3.14]	1.23 [0.47–3.14]^a^
Diabetes	36 (13.8)	32 (56.1)	8.00[4.29–15.19]**	8.10 [3.81–17.80]**^a^
BMI				
<25	110 (42.1)	17 (29.8)		
25–29.9	88 (33.7)	22 (38.6)	1.62 [0.81–3.27]	1.09 [0.48–2.46]^a^
≥30	55 (21.1)	10 (17.5)	1.18 [0.49–2.70]	0.41 [0.14–1.08]^a^
Treated hypertension	111 (42.5)	39 (68.4)	2.93 [1.61–5.50]**	1.75 [0.78–3.98]^a^
Multimorbidity (≥2 diseases)	148 (56.7)	46 (80.7)	3.19 [1.64–6.74]**	1.31 [0.54–3.26]^a^
Diabetic multimorbidity (diabetes + ≥1 additional disease)	29 (11.1)	31 (54.4)	9.54 [5.02–18.46]**	10.46 [4.87–23.35]**^b^
Non-diabetic multimorbidity (≥2 diseases)	119 (45.6)	15 (26.3)	0.43 [0.22–0.79]*	0.26 [0.12–0.56]**^b^
Non-diabetic multimorbidity (≥3 diseases)	64 (24.5)	8 (3.5)	0.50 [0.21–1.06]	0.31 [0.11–0.74]*^b^

^†^1 missing data ^‡^2 missing data. *p-value < 0.05 **p-value ≤ 0.001. ^*a*^Adjusted on sex, age, BMI, treated hypertension, diabetes, and multimorbidity. ^*b*^Adjusted on sex, age, BMI, treated hypertension.

Further analysis conducted in the multivariate models confirmed the major association between diabetes and appropriate statin prescription above multimorbidity status (Supplementary material).

Appropriate statin prescription was also more likely to be found in patients between 65 and 74 years old in multivariate models (*a*OR 2.45[1.09–5.64]), whereas being over 75 years old was not significantly associated with appropriate statin prescription.

## 4. Discussion

### 4.1. Key findings

As expected, statin therapy was under-prescribed among patients with an indication for primary prevention. There seems to be distinct categories of multimorbidity, as diabetic multimorbidity was strongly associated with appropriate statin prescription, while non-diabetic multimorbidity lowered the odds of being prescribed a statin for primary prevention in accordance with the guidelines.

### 4.2. Comparison with the existing literature

Our work raises questions about multimorbidity and how it might be dealt with by GPs in primary prevention contexts.

Among cardiovascular risk factors, diabetes appears to be the most significant factor taken into consideration. In the literature, a history of diabetes is found to be associated with higher odds of having a lipid screening, and with higher odds of appropriate statin prescription (14, 26), even though diabetic patients are also affected by statin under-prescription, both in our study and in two successive European surveys (27, 28). Our results stress that in comparison with other identified cardiovascular risk factors, diabetes seems to have a lot more weight in the prescription decision process. This might be explained by the fact that diabetic patients’ cardiovascular risk may be considered moderate to high without using any assessment scores according to the European guideline (29). This in turn might make their eligibility for statin therapy in primary prevention more striking for practitioners, especially with regard to multimorbid patients.

Our findings in adjusted models underline the need to consider cardiovascular risk factors as confounding variables. Not only are they highly prevalent in multimorbid patients (7, 8), but when we calculated odds ratios adjusted for age, sex and the presence of treated hypertension, diabetes and body mass index (BMI), multimorbidity was no longer a determining factor for statin prescription for primary prevention purposes by itself. Unlike the diabetic patients, whether multimorbid or not, non-diabetic multimorbid patients are less likely to be prescribed an appropriate preventive statin therapy.

This underlines the fact that when it comes to primary prevention and cardiovascular diseases, considering multimorbidity as a homogeneous condition is overly simple and should be complemented by further detailed descriptive analyses. At a basic level, it would certainly be beneficial to differentiate between cardiovascular multimorbidity and non-cardiovascular multimorbidity as proposed by Déruaz-Luyet et al. (22). A recent Delphi-consensus study also proposed to use weighted measures of multimorbidity when focusing on risk adjustment (6).

There is only limited evidence of GPs clinical reasoning in situations of multimorbidity, particularly regarding the prioritization of health issues. Our findings question how risk assessment scores are used in daily practice and to what extent they influence prioritization in the context of multimorbidity. Liew et al. (15) found that GPs face difficulties in determining the practical implications of cardiovascular risk calculations, especially in patients already treated for risk factors, and this may be related to an “*understanding of the limited power of any risk score to make an individual prediction of risk highlighted in some guidelines*.” These difficulties could interfere with a common prioritization strategy, that consists in ranking health problems according to their impact on morbimortality (8).

We can hypothesize that it is not just cardio-vascular multimorbidity nor the global cardiovascular risk assessment, but rather the presence or absence of diabetes, that prevails in GPs decision-making about statin prescription for primary prevention (Table 3 and Supplementary material). Differentiating between cardiovascular and non-cardiovascular multimorbidity could be a very pragmatic tool to guide the decision-making process in complex multimorbid situations. It could also constitute good-practice recommendations, with important clinical relevance (30), especially for non-diabetic multimorbid patients.

### 4.3. Strength and limitations

Despite a recruitment in only one region in France, several epidemiological data support the generalizability of our results. Indeed, the population structure in Rhône-Alpes is comparable to the nation-wide data in terms of sex-ratio (31) and age repartition (32). Though the standardized mortality rate above 65 years old is lower in Rhône-Alpes (37.5 vs. 38.4 ‰ for national data), main causes of death are comparable to national data, and cardiovascular diseases represent the second cause of death among people aged 65 and older (25% of deaths). Differences in standardized mortality rate could be explained by health disparities related to social determinants of health, as there is an important rate of intermediate occupations (33). Moreover, the rates of patients on statin medication and prevalence of multimorbidity at the time of inclusion are consistent with other epidemiological data in France and more generally in Europe (12, 23, 34). This suggests that our study population was comparable to other primary care populations across Europe. We are confident that our methodological choices in terms of GP sampling and prospective recruitment of participants strengthened the quality of the collected data. The higher appropriate statin prescription rates reported in American studies (14, 26) could be explained by the different risk assessment scores and guidelines applied there.

Our approach to multimorbidity is another strength of our study. (i) We used a robust tool to assess the presence of multimorbidity in primary care; (ii) our multivariate model included confounding variables as cardiovascular risk factors such as diabetes or treated hypertension; and (iii) we tested several categories of multimorbidity, which sharpened our analysis. Statin under-prescription in our sample is consistent with the existing literature (12).

We did not reach the target sample size. Recruitment and retention of physicians is an important issue for primary care research (35, 36). However, the assumption supporting the sample size estimation, based on a hospital sample, overestimated the rate of appropriate prescription. We found much lower appropriate statin prescription rate in our work (22.5 vs. 62%). This is not without consequences, as the closest to 50% the rate is, the larger the needed sample size is. Thus, we can assume that our final sample size might be sufficient and despite a smaller sample size than expected, our work provides insights into the associations between multimorbidity and appropriate statin prescription for primary prevention. As we found large, significant associations, particularly in relation to diabetes, we think our results remain valid. Yet, we may have missed smaller associations.

### 4.4. Implications for research and/or practice

Our work highlights the fact that in GPs’ prescription habits for cardiovascular prevention in a context of multimorbidity, diabetes weighs more than multimorbidity (7, 37). Indeed, our findings reveal that within the term multimorbidity, the presence or absence of diabetes leads to opposite associations with our variable of interest. Our results underline the necessity to consider multimorbidity as a heterogeneous entity, especially when defining the outcomes of a study in the field of cardiovascular prevention.

Additional research exploring GPs’ decision-making processes regarding the prescription of statin therapy for primary prevention purposes would help further understand and explain our findings, especially with regard to how they weigh different types of multimorbidity and cardiovascular risk levels. Our main outcome is based on appropriateness of statin prescription according to guidelines. However, as we underlined before, GPs are often confronted to a poor applicability of single-disease based guidelines in multimorbid patients (2), thus questioning the possible gap between guideline-recommended and clinically-relevant prescription. Defining diabetic multimorbidity as an entity, and differentiating between cardiovascular and non-cardiovascular multimorbidity situations may be a pragmatic way for GPs to improve primary prevention in a patient-centered and shared decision-making approach, for example through an integrated care model. Integrated care is particularly interesting in multimorbidity as it help avoid fragmentation of care, especially for patients with multimorbidity (38). First, differentiating between cardiovascular and non-cardiovascular multimorbid patients could help GPs and/or advanced nurse practitioners involved in multimorbid patients’ care (39) to identify those most likely to need an extended cardiovascular risk assessment. An alert integrated in the medical record software could help increase practitioners’ vigilance. Presence of diabetes in itself could also be a trigger, independently of associated chronic diseases as most diabetic patients have blood pressure issues or other risk factors, and are for the most part at high risk (27). Cardiovascular risk assessment guided by validated scores could be conducted by a medical assistant and registered in the shared medical electronic record. If high cardiovascular risk is confirmed, an automated alert could then warn the GP and/or the advanced practice nurse that a consultation should be organized and dedicated to discussing with the patient his cardiovascular risk level and the interventions that would be appropriate, in order to reach a shared understanding and agreement about the prescriptions that the GP could initiate. Not only advanced nurse practitioners could have the competencies to help better manage cardiovascular risk in multimorbid patients (39) but acceptability of coordinated care models between GPs and advance nurse practitioners seems high (40). Last, but not least, integrated care models also help improve both health outcomes–for example in patients with diabetes- and cost effectiveness of care (41, 42).

## Data availability statement

The raw data supporting the conclusions of this article will be made available by the authors, without undue reservation.

## Ethics statement

Ethical review and approval was not required for the study on human participants in accordance with the local legislation and institutional requirements. The patients/participants provided their written informed consent to participate in this study.

## Author contributions

All authors listed have made a substantial, direct, and intellectual contribution to the work, and approved it for publication.
