# Supplementary material for: Multimorbidity and statin prescription for primary prevention of cardiovascular diseases: A cross-sectional study in general practice in France
Source: Front Med (Lausanne). 2023 Jan 9;9:1089050. doi: 10.3389/fmed.2022.1089050 (PMC9868625; doi:10.3389/fmed.2022.1089050)
Supplement: Supplementary file 1 [file Data_Sheet_1.PDF]

Supplementary Material

Supplementary Table 1: characteristics of participating general practitioners

	N = 40	%
Men	19	48
Age		
- 25-34	8	20
- 35-44	18	45
- 45-54	6	15
- 55-64	7	17.5
- 65-74	1	2.5
Urban practice	25	62.5
Group practice (pluriprofessional or monoprofessional)	36	90
Participation in workplace-based learning for residents	22	55
Continuing medical education	39	97.5

Supplementary Table 2: Factors associated with statin prescription – model 1

	No statin therapy (N = 261)	Appropriate statin therapy (N = 57)	OR [95CI] (univariate)	aOR[a]
Female	163 (62.5)	27 (47.4)	0.54 [0.30-0.96] ; p = 0.037	0.58 [0.29-1.17] ; p = 0.128
Age				
- < 65	135 (52%)	19 (33.3)		
- 65-74	70 (27)	27 (47.4)	2.74 [1.43-5.34] ; p=0.003	**2.45 [1.09-5.64] ; p = 0.032**
- ≥ 75	55 (21)	11 (19.3)	1.42 [0.62-3.14] ; p = 0.393	1.23 [0.47-3.14] ; p = 0.672
Diabetes	36 (13.8)	32 (56.1)	8.00 [4.29-15.19] ; p < 0.001	**8.10 [3.81-17.80] ; p < 0.001**
BMI				
- < 25	110 (42.1)	17 (29.8)		
- 25 – 29.9	88 (33.7)	22 (38.6)	1.62 [0.81-3.27] ; p = 0.173	1.09 [0.48-2.46] ; p = 0.830
- ≥ 30	55 (21.1)	10 (17.5)	1.18 [0.49-2.70] ; p = 0.706	0.41 [0.14-1.08] ; p = 0.080
Hypertension under medication	111 (42.5)	39 (68.4)	2.93 [1.61-5.50] ; p = 0.001	1.75 [0.78-3.98] ; p = 0.175
Multimorbidity (≥ 2 diseases)	148 (56.7)	46 (80.7)	3.19 [1.64-6.74] ; p = 0.001	1.31 [0.54-3.26] ; p = 0.550

[a]*Adjusted on sex, age, diabetes, BMI, treated hypertension and multimorbidity*

Supplementary Table 3: Factors associated with statin prescription – model 2

	No statin therapy (N = 261)	Apropriate statin therapy (N = 57)	OR [95CI] (univariate)	aOR[c]
Female	163 (62.5)	27 (47.4)	0.54 [0.30-0.96] ; p = 0.037	0.55 [0.27-1.12] ; p = 0.101
Age				
- < 65	135 (52%)	19 (33.3)		
- 65-74	70 (27)	27 (47.4)	2.74 [1.43-5.34] ; p=0.003	**2.45 [1.08-5.67] ; p = 0.033**
- ≥ 75	55 (21)	11 (19.3)	1.42 [0.62-3.14] ; p = 0.393	1.35 [0.51-3.48] ; p = 0.538
BMI				
- < 25	110 (42.1)	17 (29.8)		
- 25 – 29.9	88 (33.7)	22 (38.6)	1.62 [0.81-3.27] ; p = 0.173	1.04 [0.46-2.35] ; p = 0.919
- ≥ 30	55 (21.1)	10 (17.5)	1.18 [0.49-2.70] ; p = 0.706	0.38 [0.13-1.05] ; p = 0.070
Hypertension under medication	111 (42.5)	39 (68.4)	2.93 [1.61-5.50] ; p = 0.001	1.58 [0.74-3.40] ; p = 0.242
Diabetic multimorbidity (diabetes + ≥ 1 additional disease)	29 (11.1)	31 (54.4)	9.54 [5.02-18.46] ; p < 0.001	**10.46 [4.87-23.35] ; p < 0.001**

[c]*Adjusted on sex, age, BMI, treated hypertension and diabetic multimorbidity*

Supplementary Table 4: Factors associated with statin prescription – model 3

	No statin therapy (N = 261)	Apropriate statin therapy (N = 57)	OR [95CI] (univariate)	aOR[d]
Female	163 (62.5)	27 (47.4)	0.54 [0.30-0.96] ; p = 0.037	0.55 [0.28-1.07] ; p=0.077†<br>0.59 [0.29-1.19] ; p = 0.139‡
Age				
- < 65	135 (52%)	19 (33.3)		
- 65-74	70 (27)	27 (47.4)	2.74 [1.43-5.34] ; p=0.003	**2.50 [1.16-5.52] ; p = 0.021†**<br>**2.47 [1.10-5.67] ; p = 0.030‡**
- ≥ 75	55 (21)	11 (19.3)	1.42 [0.62-3.14] ; p = 0.393	1.59 [0.63-3.91] ; p = 0.320†<br>1.28 [0.48-3.31] ; p = 0.609‡
Diabetes	36 (13.8)	32 (56.1)	8.00 [4.29-15.19] ; p < 0.001	**8.05 [3.28-21.02] ; p < 0.001‡**
BMI				
- < 25	110 (42.1)	17 (29.8)		
- 25 – 29.9	88 (33.7)	22 (38.6)	1.62 [0.81-3.27] ; p = 0.173	1.07 [0.50-2.31] ; p = 0.857†<br>1.06 [0.47-2.38] ; p = 0.890‡
- ≥ 30	55 (21.1)	10 (17.5)	1.18 [0.49-2.70] ; p = 0.706	0.78 [0.30-1.92] ; p = 0.590†<br>0.42 [0.15-1.12] ; p = 0.093‡
Hypertension under medication	111 (42.5)	39 (68.4)	2.93 [1.61-5.50] ; p = 0.001	**2.97 [1.43-6.37] ; p = 0.004†**<br>1.98 [0.91-4.40] ; p = 0.088‡
Non-diabetic multimorbidity (≥ 2 diseases)	119 (45.6)	15 (26.3)	0.43 [0.22-0.79] ; p = 0.009	**0.26 [0.12-0.56] ; p = 0.001†**<br>0.88 [0.33-2.34] ; p = 0.795‡

[d]*Adjusted on sex, age, BMI, treated hypertension, nondiabetic multimorbidity (≥ 2), without† or with‡ diabetes*

Supplementary Table 5: Factors associated with statin prescription – model 4

	No statin therapy (N = 261)	Apropriate statin therapy (N = 57)	OR [95CI] (univariate)	aOR[e]
Female	163 (62.5)	27 (47.4)	0.54 [0.30-0.96] ; p = 0.037	**0.52 [0.27-1.01] ; p = 0.053†**<br>0.59 [0.29-1.19] ; p = 0.139‡
Age				
- < 65	135 (52%)	19 (33.3)		
- 65-74	70 (27)	27 (47.4)	2.74 [1.43-5.34] ; p=0.003	**2.64 [1.24-5.74] ; p = 0.013†**<br>**2.49 [1.11-5.72] ; p = 0.028‡**
- ≥ 75	55 (21)	11 (19.3)	1.42 [0.62-3.14] ; p = 0.393	1.59 [0.64-3.87] ; p = 0.310†<br>1.31 [0.49-3.39] ; p = 0.580‡
Diabetes	36 (13.8)	32 (56.1)	8.00 [4.29-15.19] ; p < 0.001	**8.03 [3.65-18.38] ; p < 0.001‡**
BMI				
- < 25	110 (42.1)	17 (29.8)		
- 25 – 29.9	88 (33.7)	22 (38.6)	1.62 [0.81-3.27] ; p = 0.173	1.21 [0.57-2.56] ; p = 0.624†<br>1.06 [0.47-2.38] ; p = 0.885‡
- ≥ 30	55 (21.1)	10 (17.5)	1.18 [0.49-2.70] ; p = 0.706	0.89 [0.35-2.19] ; p = 0.804†<br>0.43 [0.15-1.15] ; p = 0.101‡
Hypertension under medication	111 (42.5)	39 (68.4)	2.93 [1.61-5.50] ; p = 0.001	**2.30 [1.14-4.72] ; p = 0.021†**<br>1.96 [0.93-4.24] ; p = 0.080‡
Nondiabetic multimorbidity (≥ 3 diseases)	64 (24.5)	8 (3.5)	0.50 [0.21-1.06] ; p = 0.091	**0.31 [0.11-0.74] ; p = 0.015†**<br>0.79 [0.26-2.13] ; p = 0.655‡

[e]*Adjusted on sex, age, BMI, treated hypertension, nondiabetic multimorbidity (≥ 3), without† or with‡ diabetes*

Supplementary Table 6: Indications of statin therapy for primary prevention in the HAS 2017 recommendation

Cardiovascular risk level (SCORE)	LDL-c level targeted	First line intervention	Second line intervention
Low risk<br>Moderate risk	< 190 mg/dL<br>< 130 mg/dL	Lifestyle modification	Lifestyle modification + statin therapy
High risk<br>Very high risk	< 100 mg/dL<br>< 70 mg/dL	Lifestyle modification + statin therapy	Lifestyle modification + intensification of statin therapy
